# Keynotes, and the Totality of the Symptoms

**Published:** 1887-02

**Authors:** E. W. Berridge

**Affiliations:** London


### KEYNOTES, AND THE TOTALITY OF THE SYMPTOMS.

E. W. Berridge, M. D., London.

The International Hahnemannian Association has very properly declared one of its principles to be "the *totality* of the symptoms as the only basis for prescribing." This is in full accordance with Hahnemann's teaching in section 7 of his *Organon*, where he says that "the *totality of the symptoms* must be the principal, the sole thing, the physician has to take note of in *every* case of disease;" and again (section 18), "the sum of all the symptoms in *each* individual case of disease must be the sole indication, the sole guide, to direct us to the choice of a curative remedy." Further on, in section 153, he recognizes the fact that some symptoms are of more diagnostic value in the selection of the remedy than others; stating that "the more striking, singular, uncommon, and peculiar (characteristic) sign and symptoms of the case of disease are *chiefly* and *almost* solely to be kept in view; for it is *more particularly* these that very similar ones in the list of symptoms of the medicines sought for must correspond to. * * * The more general and undefined symptoms, loss of appetite, headache, debility, restless sleep, discomfort, and so forth, demand *but little* attention when of that vague and indefinite character, if they cannot be more accurately described, as symptoms of such a general nature are observed in almost every disease and from almost every drug."

Here Hahnemann's teaching is plain; the *totality* of the symptom is *always* to be regarded, the more characteristic symptoms especially so, but even the less characteristic must not be altogether neglected.

This Hahnemannian doctrine was also the doctrine of the late Professor H. N. Guernsey, the so-called "father of the keynote system," whose words on this point have been perverted from their true meaning. Though he gave in his *Obstetrics* and in the *Hahnemannian Monthly* a large number of characteristics or "keynotes," he never meant that they should form the *sole* basis of a prescription, which would indeed be simply prescribing for a single symptom. On the contrary, he declared (*New England Medical Gazette*, 1877, p. 120), "All characteristics or keynotes must be in harmony with and be confirmed by the totality of the accompanying symptoms before we have sufficient reason for prescribing." The same teaching is given in the preface to the first edition of his *Obstetrics*, published in 1873. "It may seem like prescribing for single symptoms, whereas such is not the fact; it is only meant to state some strong characteristic symptom, which will often be found the governing symptom, and on referring to the *Symptomen Codex* all the others will surely be there if this one is;" that is, as the author says a few lines further on, "If that remedy is well proven."

Surely these extracts should suffice to show that to prescribe for single symptoms is neither what was meant by Hahnemann when he wrote of characteristics, nor by Guernsey when he taught the use (not the abuse) of keynotes. But should anything be needed in addition to demonstrate not only the unscientific character of such prescribing, but its absolute impracticability, I will quote from page 7 of the preface to Hering's *Guiding Sgmptoms:* "The definition of a characteristic being a symptom not found under more than one remedy" is quite erroneous. "All our most approved characteristics, as they have been corroborated time and time again, are never such as are found in one medicine alone."

Yet in spite of all these teachings from our veterans, there has been a tendency on the part of some recent converts to attempt a short cut to Homœopathy, a sort of "see-the-case-at-a-glance" prescription, by means of an *exclusive* reliance on supposed characteristics, to the neglect of the rest of the symptoms. Thus, a few years ago, a writer asked: "Is every symptom of *Sulphur* as narrated in Allen's *Encyclopædia* a pure symptom of the drug and to be used as an indication for its therapeutic use? I know not! I thought it was the *characteristic* symptoms which were the guiding symptoms, and not 'every' symptom." Whether every symptom attributed to *Sulphur* is reliable has not hitherto been demonstrated, but assuredly every reliable symptom is to be used as an indication for treatment, or else Hahnemann and Guernsey and the I. H. A. are sadly mistaken!

It is, I believe, the fatal error of prescribing for single symptoms which led to Lux's heresy of Isopathy, or the prescribing of a dynamized nosode for every case of disease with a corresponding name—a practice which is completely at variance with the Hanemannian and *scientific* method of prescribing nosodes, and all other remedies, according to the totality of the symptoms. To give an example, I never prescribe *Syphilinum* because I have diagnozed the case as either acute or chronic syphilis. I prescribe it because the symptoms of the individual patient indicate this remedy according to reliable provings or clinical observations; and in such a case I prescribe it without troubling myself (so far as the selection of the remedy is concerned) whether the disease is chiefly syphilitic or only complicated with syphilis, or neither. Yet we were told, only a few years ago, that "before any one could select *Syphilinum* with safety and with certainty, he must first be able to recognize syphilis in all its forms, and be able to differentiate between it and psora."

With all due deference to the writer (who, I hope, is wiser now), I maintain, first, that such a method of prescribing is non-homœopathic; and, secondly, that if it were homœopathic it would be impracticable, as the diagnosis cannot always be established, not even by the skill of the Great Infallible himself, who not very long ago arrogantly and insolently declared of us, that, "so far as my experience of homœopathicians is concerned, I mean of the so-called Hahnemannians, they are, as a rule, most miserable hands at physical diagnosis."

With these prefatory remarks, I will now quote a few of the so-called keynotes, and prove that a reliance on them, *apart from the totality of the symptoms*, must prove a mockery, a delusion, and a snare.

(1) In the August number of the *Medical Advance*, Dr. Skinner writes a strong and much needed protest against the local treatment of leucorrhœa.* At p. 132 he says of the symptom,

---

* I must, however, here express my want of concurrence with Dr. Skinner's pathological doctrines as given in this article, though, of course, I fully agree with his denunciation of local treatment. He makes these four assertions:

(1) That the doctrine that "no diseased action could proceed without tissue change is simply nonsense and not fact."

(2) That the doctrine that "no discharge could take place from a mucous surface without inflammatory action" is ditto.

(3) That the vagina is the "constitutional waste-pipe peculiar to the female bodily and mental constitution."

(4) That "leucorrhœa" is a healthful discharge. How can it be healthful if it is a disease? Were it inflammatory, like dysentery, it would be disease. But it is no more inflammatory than defecation, which it is to be presumed is a healthful, healthy, and health-giving process.

cutting pains in abdomen from right to left, "One dose of *Lycopod.* high, and the higher the better, will give a good account of the leucorrhœa and its homœopathic relation to *every case of leucorrhœa where that concomitant is present.*" Undoubtedly this is a valuable symptom; but *Nux moschata* (another remedy for leucorrhœa) has also produced a similar symptom, and there must surely therefore be some cases in which it, and not Lycop., is indicated. *Ipecac.* has a similar pain from left to right.

(2) Stool passes better when standing: *Causticum.* This is another characteristic symptom which I have twice verified; but it has also been cured with *Aluminia* $^{30}$. *Medorrhinum* has the similar symptom, "Can only pass stool by leaning the body very far back." (N. B.—It is *not* necessary always to diagnose suppressed gonorrhœa before prescribing this nosode should this symptom be met with in practice. The totality of the symptoms will decide the choice.)

(3) Emptiness in stomach at eleven A.M.: *Sulphur.* This has been quoted as a keynote, but it is not infallible. The symptom also belongs to *Hydrastis, Lachesis, Natr.-phos.*, and *Zinc.* I was recently consulted by a colleague, one of our rising men, in a case of chronic constipation, where this symptom existed. *Sulphur* $^{200}$ had been given with only temporary and partial relief. I selected *Lachesis*, according to the totality of the symptoms, and the effect was remarkable.

(4) *Feeling of damp stockings on the feet: Calcarea-carb.* This must not always be relied on as an indication for *Calc.*, as it is found also under *Saponinum*, which has also a similar feeling on the hands, the effect of one of the above eight remedies, carefully selected *according to the totality of the symptoms.*

(5) One foot hot and the other cold. This symptom is often quoted as a keynote of *Lycop.* The original states, "coldness of right foot with heat of left," which I have verified; but as

---

I would suggest in opposition to these views:

(1) That no diseased action, even what is called purely functional, can possibly take place without molecular tissue-change.

(2) That no morbid condition has yet been demonstrated to produce abnormal reaction from mucous surfaces, except some form of inflammation, or at least congestion, which is its preliminary stage.

(3) That how the *mind* can have a bodily "waste-pipe" is not self-evident to an ordinary comprehension like my own.

(4) That if leucorrhœa is "healthful" it ought not to be removed by any means; and if analogous to defecation, no woman (as a rule) can be healthy unless she suffers from a leucorrhœal discharge about once in the twenty-four hours.

*Quod est absurdum.*

Bœnninghausen quotes it, "heat of one foot, the other cold," I conclude that he found by clinical experience that in this symptom the side did not matter. But the symptom also belongs to Hura brasiliensis.

(6) Movements like the kick of a child. This symptom Dr. Skinner verified in a recently published case of spurious pregnancy, and as he claims it is the only remedy which has this symptom, I conclude he considers it an infallible keynote. Yet it has been produced by *Convallaria majalis, Crocus, and Thuja*, while *Carlsbad* has produced "sensation like the first movements of a child," added to which, in *The Organon*, Vol. III, p. 270, is quoted a case of false pregnancy cured by *Pulsat.* Dr. Swan informed me that he had seen the symptoms thereof removed with Lac vaccinum defloratum, and Dr. Kent writes that he has cured the very symptom quoted with *Sepia.*

